# Process and impact evaluation of the Greater Christchurch Urban Development Strategy Health Impact Assessment

**DOI:** 10.1186/1471-2458-9-97

**Published:** 2009-04-05

**Authors:** Kaaren R Mathias, Ben Harris-Roxas

**Affiliations:** 1 Community and Public Health, Canterbury District Health Board, Christchurch, New Zealand; 2 Centre for Health Equity Training, Research and Evaluation, part of the Centre for Primary Health Care and Equity, School of Public Health and Community Medicine, University of NSW, Sydney, Australia

## Abstract

**Background:**

despite health impact assessment (HIA) being increasingly widely used internationally, fundamental questions about its impact on decision-making, implementation and practices remain. In 2005 a collaboration between public health and local government authorities performed an HIA on the Christchurch Urban Development Strategy Options paper in New Zealand. The findings of this were incorporated into the Greater Christchurch Urban Development Strategy;

**Methods:**

using multiple qualitative methodologies including key informant interviews, focus groups and questionnaires, this study performs process and impact evaluations of the Christchurch HIA including evaluation of costs and resource use;

**Results:**

the evaluation found that the HIA had demonstrable direct impacts on planning and implementation of the final Urban Development Strategy as well as indirect impacts on understandings and ways of working within and between organisations. It also points out future directions and ways of working in this successful collaboration between public health and local government authorities. It summarises the modest resource use and discusses the important role HIA can play in urban planning with intersectoral collaboration and enhanced relationships as both catalysts and outcomes of the HIA process;

**Conclusion:**

as one of the few evaluations of HIA that have been published to date, this paper makes a substantial contribution to the literature on the impact, utility and effectiveness of HIA.

## Background

Health impact assessment (HIA) has been promoted for over twenty years as a way to enhance the potential effects of a policy or strategy "on the health of a population, and the distribution of those effects within the population " [1]. HIA uses a mixture of quantitative and qualitative predictive methods and community consultation to identify, and where possible quantify, a specific proposal's direct and indirect impacts on health [2]. Impact assessment has been used in New Zealand in the environmental field for many years [2-5]. More recently HIA has been promoted to assess the impacts of national and local policy on health, wellbeing and equity. The New Zealand Health Strategy includes HIA of policy under Objective One [6]. HIA particularly seeks to consider the impact of policy on health inequalities.

Although the term 'health inequalities' is relatively new, the effect of urbanisation to disadvantage the poor was noted over one hundred and fifty years ago. Urban planning plays an important role in shaping the environmental, social and economic health determinants in cities [7-9].

Evaluations of Health Impact Assessments have been infrequently performed, and rarely published. Evaluation of HIA has been identified as the field of HIA research most urgently requiring attention [3,10-15]. With HIA's use becoming more widespread, it is important to describe what HIA can achieve and what it can not.

The Greater Christchurch Urban Development Strategy HIA (UDS HIA) provided an opportunity for both a process and an impact evaluation. Process and impact evaluations allowed review of the HIA itself and its impacts on policy over a medium term time frame (two to three years). While outcome evaluation to determine validity of HIA predictions and changes in health and health determinants is the most desirable measure of success of an HIA, in reality the 20 – 30 year time frame required and numerous factors that impact on health outcomes make it difficult if not impossible to perform. The process evaluation was undertaken concurrently with the HIA being conducted in 2005, while the impact evaluation was undertaken in 2008.

This report adds to the limited existing literature on evaluation of HIA. The process evaluation sought to answer whether this HIA achieved its objectives, identify success factors and to quantify resources use. The impact evaluation's objectives were to describe the impacts of the HIA on the final UDS, including possible reasons for inclusion, describe unintended impacts of the HIA and evaluate the effectiveness of HIA in policy. Points are discussed in detail and several conclusions made for future evaluations of HIAs.

### HIA in New Zealand

In New Zealand central, regional and local government each have a role in urban development and planning in New Zealand. The Regional Council has responsibility for preparing the Regional Policy Statement under the Resource Management Act. This sets the direction for managing the region's natural resources and outlines the settlement pattern for a region. Local councils must give effect to the Regional Policy Statement through their District Plans which provide the framework for the management of land use and subdivision.

Incorporation of Treaty of Waitangi principles is implicit in HIA in New Zealand [5]. HIA specifically examines possible impacts of policy on Māori and to ensure that policy formation follows Treaty of Waitangi principles of participation, partnership and protection.

### Greater Christchurch Urban Development Strategy HIA 2005

The Greater Christchurch UDS is an initiative in the Canterbury region of New Zealand, involving local government authorities (Christchurch City Council (CCC), Waimakariri and Selwyn District Councils, and Environment Canterbury (the Regional Council) and Transit New Zealand (now the New Zealand Transport Agency). The Strategy seeks to guide urban growth in the Greater Christchurch region over the next forty years with predictions that the region's current population of 380000 will have grown to 500000 [16].

In April 2005, a public consultation document on options for growth and development in the greater Christchurch region was made public. It summarised key issues and presented three options for managing growth as: concentration, consolidation and dispersal versus the 'business as usual' option (where urban growth is less regulated and driven by private developer demand). Of 3250 feedback forms received, 62% supported the Concentration option. This focussed 60% of new housing in urban renewal and 40% in new land release subdivisions.

In June 2005 Community and Public Health, a division of the Canterbury District Health Board (CDHB) proposed to CCC staff that they perform a Health Impact Assessment on the options document of the Greater Christchurch UDS [16]. The CCC planning and policy team responsible for the UDS policy formation supported and welcomed an HIA from the first scoping and screening workshop, expressing interest in using HIA findings within the strategy. They accommodated HIA timelines within the strategy project requesting HIA completion (including a presentation to the Urban Forum) by December 2005. The UDS team worked collaboratively with CDHB staff to optimise the HIA process. Key stakeholders of the HIA steering group were CDHB, CCC and Environment Canterbury. The project working group comprised two public health physicians from CDHB and two senior policy analysts from CCC. One public health physician was the HIA project leader.

The specific aims and objectives of the HIA were:

I. To provide evidence about the links between urban development and health for decision-making;

II. To assess the positive and negative health impacts of the UDS and provide recommendations to increase positive and decrease negative inputs;

III. To strengthen partnerships working between sectors and ensure appropriate participation of the community including those that are vulnerable due to social exclusion;

IV. To involve Māori in all levels of the HIA process; and

V. To build capacity and knowledge of HIAs in Christchurch and New Zealand.

Interestingly the HIA did not explicitly include any aims and objectives relating to maximising the extent and distribution of positive health impacts and mitigating negative health impacts, though this may be because these objectives were regarded as axiomatic of all HIAs. The HIA followed the standard HIA process of screening, scoping, appraising and evaluation [5]. An initial screening and scoping workshop was held, and was later found to be critical to engaging key stakeholders and ensuring the future collaboration between local government and health as partners, both in the HIA and subsequent projects. The HIA model was pragmatic, prospective and used multiple agencies and disciplines. This 'fit for purpose' approach to HIA [17,18] seeks to deliver the benefits of the HIA within existing resource and time constraints. The HIA therefore involved rapid appraisals and no primary research. The HIA methodology included:

• Screening the UDS to determine if it was suitable for an HIA to be conducted;

• Scoping the HIA;

• Eight rapid appraisal workshops in technical areas (two to four hours each);

• Literature reviews and summaries

• Report-back to workshop participants via the Internet and a summary meeting;

• Circulating the draft HIA report to key stakeholders, then presenting it to the UDS Management Team and Forum [19]

• Concurrent process evaluation.

Scoping led to a focus on six determinants of health: air, waste, water, social connectedness, housing and transport. Waste was subsequently omitted due to resource constraints. A further working group was developed around engaging with local Māori who were significantly under-represented in the UDS consultation process. Only 1.5% of the 3250 respondents to the UDS Options document were Māori, yet Māori make up 7.3% of the population of Canterbury and have the poorest health status of any ethnic group in New Zealand [20]. The UDS HIA has been reported on in greater detail elsewhere [21-23].

## Methods

The process and impact evaluations followed published evaluation methodologies [14,15,24]. An employee of CDHB acted as the HIA evaluator. Risks of loss of independence and bias were managed through review of methods and findings with colleagues at the School of Medicine at the University of Otago, Christchurch, and CCC at key points through the evaluation process. It was undertaken in collaboration with the HIA working group who worked with the evaluator to develop the evaluation objectives and methods and assisted in data collection.

Process and impact evaluations used multiple qualitative research methodologies (key informant interviews, focus groups, workshops, surveys and document review) as primary methods of data gathering to maximise data richness and understanding. Findings were analysed thematically and triangulated using different methods to examine the same issue, to increase the reliability of findings. Greater detail on methods and all aspects of the evaluations is available in the original evaluation reports [25,26].

### Process evaluation

Objectives of the process evaluation were to assess whether this HIA achieved its stated objectives, to identify critical success factors and opportunities for improvements in the process, and to quantify resource use.

Four of the eight HIA workshops were evaluated by participant observation, a review of the workshop report and HIA outputs including reports and other documentation, and surveys completed by workshop participants. A single focus group was held. The surveys of workshop participants used open-ended questions and Likert scales. The documentary analysis was undertaken on working group minutes, key reports and background documents. Resource-use was measured and hours estimated by a questionnaire filled in by project and human resources staff of CDHB. Hourly rates were based on salaries in 2005 and all comparisons with international HIA costings use US Dollars at December, 2005 exchange rates.

### Impact evaluation

The objectives of the impact evaluation were to assess what changes to decision-making and implementation had occurred as a result of doing the HIA, and what other indirect impacts had eventuated, such as changes to cross-sectoral relationships, engagement with Māori, and knowledge and understanding of HIA. Key informant interviews used a semi-structured format. The interview schedule included in Table 1 was modified for key informants outside Christchurch. The sample of key informants was identified by members of the HIA working group and based on individuals who had been involved in the design or implementation of the HIA, as well as those who had been involved in an advisory capacity. The sample of 23 included representatives of each UDS partner organisation, as well as steering group members, and New Zealand and Australian HIA consultants and academics. Three of the original 23 identified were not available. Key informants were interviewed face-to-face (and by telephone for those from outside of Christchurch) using the interview schedule in Table 1.

**Table 1:** Interview schedule for impact evaluation – Key Informants

1. From your knowledge how was the Christchurch HIA used in policy development and advice?
2. How was the UDS (as the policy proposal) changed as a result of the HIA?
3. To what extent that you are aware, were the recommendations of the HIA accepted and implemented by policy makers? Do you know of any mitigation measures undertaken?
4. What do you see as more distal impacts/'side spin-offs' of the Christchurch HIA? E.g. synergies/partnerships/increased role of health on the agenda.
5. Are there ways you can think of that this HIA did not fulfil or meet expectations? Can you elaborate?

Stakeholders from the original key informant sample, as well as a local government politician from that time, and members of the Urban Forum and local government staff involved in implementation of the UDS were provided with a briefing report summarising findings from key informant interviews and the document analysis, and invited to a full-day HIA impact evaluation workshop in April 2008. Fourteen people attended the workshop. Data was thematically analysed to identify emerging patterns. Both evaluations specifically sought to identify unintended impacts of the HIA as well as to describe aspired impacts that had not been achieved.

The document review included the final UDS [27], HIA report [21] and relevant CDHB and CCC documents. The primary output of the document review is summarised in Table 2.

**Table 2:** Summary of HIA recommendations included in the UDS Key Approaches, Actions and Top Twenty Priority Actions

**Focus of recommendations in the HIA**	**Number of HIA recommendations included in UDS Approaches**	**Number of UDS Actions addressing HIA recommendations**	**Number of Top Twenty Actions linked to HIA recommendations**
Summary	1 of 4	1	0

Air	4 of 6	2	0

Water	4 of 5	2	1

Social connectedness	6 of 6	5	2

Housing	3 of 4	2	1

Transport	5 of 7	4	0

Māori	1 of 1	1	0

**TOTAL**	**24 of 33**	**17**	**4**

## Results

Results for both the process and impact evaluation will be presented in an integrated format which synthesises findings rather than separate reporting of each qualitative methodology used. Roman numerals used in reporting results relate to relevant HIA objectives.

### Process evaluation results

Findings of the process evaluation are described against the HIA objectives (listed earlier) as it sought to evaluate whether these had been fulfilled, and in a table summarising resources used (Table 3).

**Table 3:** Summary of human and financial resources used in the Greater Christchurch UDS Health Impact Assessment

	**Time** ***(hours)***	**Cost** ***(NZD)***
**Employment costs**		

HIA meeting hours		
Formal meeting hours	364	$9100

Steering group meetings	36	$2160

HIA dedicated employed hours	950	$31850

**TOTAL **(HIA employed + meeting hours)	**1350**	**$44210**

**TOTAL **(Multiplied by 50% for overhead costs)		**$66315**

**Direct costs**		

Contractors		$3840

Literature reviewers		$1700

Flights/travel		$770

Conferences attended		$1500

Other (Catering)		$200

**TOTAL**		**$8010**

SUBTOTAL (direct costs)		$8010

SUBTOTAL (employment costs)		$66315

**GRAND TOTAL**		**$74325**

### Did the HIA fulfil its objectives?

Key stakeholders reported during the process evaluation that the HIA had fulfilled all five of its objectives. The HIA report was regarded as having provided a comprehensive literature review of evidence to support decision making. Each working group was supported by a literature review summarising the key health impacts of different urban development approaches which included literature search strategies and search terms used, and multiple citations of peer-reviewed literature [21]. The recommendations included measures to enhance potential positive health impacts and mitigate potentially negative health impacts of the UDS (II). For example, in the Air Quality section the HIA recommendation says: "Projects should aim to reduce the reliance on solid fuel burners while ensuring availability of affordable and healthy alternative heating options." The HIA strengthened partnerships and had a high level of cross-sectoral support (III). This was endorsed by steering and working group members as well as participants of workshops in questionnaire responses. Māori were involved throughout the HIA process (IV). This was illustrated through Māori participation in the workshops (screening and appraisal), *hui *(a Maori word describing a formal gathering following Maori protocols) and presentation of the UDSHIA report at the Urban Forum [28]. The UDS HIA increased HIA related capacity and knowledge regionally and nationally (V). This was not demonstrable until the time of impact evaluation and is described in that section of the results.

Most participants were very positive about involvement in the HIA and felt it facilitated new cross-sectoral relationships, was enjoyable, and met its objectives. They described the HIA as important and groundbreaking in both Canterbury and New Zealand and commented on its importance in relationship-building, particularly in the Māori working group. There was strong support for the cross-sectoral workshops with several participants in both waste and water workshops (from Environment Canterbury and Environmental Science & Research (ESR) – a Crown Research Institute) describing workshops as a first ever occasion where all the people working on that issue were seated together in one room, and considered this led to opportunities for working together. Two participants in technical workshops felt the HIA added little new information and was a poor use of their time.

Questionnaire respondents described the social connectedness workshops as particularly effective at ensuring a participatory process for the community. Three social connectedness workshops were held with many high-needs groups such as new immigrants, mental health consumers, Pacific people, and beneficiaries represented. A disabled group and representative of the Chinese association particularly described value in this workshop and felt it had increased their 'voice' for being heard in the UDS consultation process.

The primary issues identified restricting the conduct of the HIA was the time limitation (only four months) and limited human resource (equivalent to one person working for five months full time).

### Resource use

Most of the costs in Table 3 were borne by the CDHB, CCC and Environment Canterbury using existing employees and budget lines. The total costs of NZD 74325 (USD 42421 on 15 December 2005) do not include opportunity or transactional costs.

### Impact evaluation findings

This section details whether HIA objectives were met, impacts on policy, impacts of individual HIA objectives, indirect and aspired impacts and ideas for future directions of HIA in Canterbury.

The final HIA report was presented to the Greater Christchurch Urban Forum in November 2005 and published in April 2006 [21]. CCC's commitment to the HIA was shown by their publication of a four page summary of the HIA [29].

### Policy changes resulting from HIA (HIA Objectives I and II)

The final UDS Strategy [27] includes many policy components that were recommended in the HIA as well as several that were not. Whilst the inclusion of components recommended in the HIA cannot be attributed solely to the HIA, their inclusion indicates an acceptance of the important role the UDS will have in determining future population health outcomes in the Canterbury region. Key informants agreed that the health content of UDS increased significantly after the UDS HIA report.

The UDS document is nearly 200 pages long with an initial background and context section. It describes the Top Twenty Priority Actions for the next three years to ensure necessary governance structures and implementation frameworks (p34–36) [27]. This is followed by the body of the strategy, with six Strategic Direction Areas and their implementation actions. For example, the 'Enrich Lifestyles' Strategic Direction Area, has within it areas such as health and wellbeing, education, community development. Each implementation action area covers four aspects:

• Explanation (why an action is being undertaken);

• Growth issues (summary of issues identified in consultation process);

• Key approaches (policy approaches to be taken that will guide action implementation);

• Actions – which use a tabular format and cover Action, Lead agency, Support agencies, Cost implications, Implementation tools, Links to strategy and Timing.

The process of assessing and incorporating HIA recommendations into the UDS was described by key informants as informal. Responsibility for decision-making rested with planning and policy making staff at CCC responsible for the UDS. They described having read and considered the HIA report while preparing the policy document. In some sections the HIA recommendations were directly translated into UDS 'policy approaches' but not necessarily into the action tables. For example, in the transport section the HIA recommended "Minimise adverse effects on communities when constructing and developing arterial roads (p 50) [21]. The UDS Key Approach corresponding with this advised: "Avoid severing communities from facilities by major highways." (p53) [27]. This had no linked action. Key informants within the UDS team confirm that while only the action tables are being used for monitoring implementation of the UDS, the policy approaches define the underlying philosophy and will continue to direct urban development in subsequent revisions of the UDS. Table 2 summarises the extent of inclusion of HIA recommendations in the final UDS document.

#### Overall section on Health and Well-being

A key impact of the HIA within the final UDS was the inclusion of a new section in the final Strategy titled "Health and Well-being" authored by the HIA project leader. This section includes an explanation of Health's inclusion in the strategy. Health indicators, however, were not mentioned in Priority Action Number Thirteen where other similar indicators were located.

### Summary of HIA recommendations and inclusion in the UDS

#### General recommendations

Of four general recommendations, only the one recommending that HIA is incorporated into the development and analysis of UDS policy was included in the final UDS.

### Air quality

four of six recommendations were included in the UDS, but only two translated into action points. A recommendation on the need for cross-sectoral collaborative working groups was not included.

#### Water quality

four of five recommendations, with two related action points, were included in the UDS. The HIA recommendation advocating for a cross-sectoral steering group for water resource management in the region, with Ngāi Tahu (the Māori tribe or 'iwi' who are 'tangata whenua' (people of the land) in Canterbury) representation, was not included in the UDS.

#### Social connectedness

all six HIA recommendations with five linked action points were included in the UDS. The Top Twenty Priority Actions also included two generic actions around social connectedness (Numbers Sixteen and Nineteen).

#### Housing

three of four HIA recommendations with two action points were included.

#### Transport

five of seven HIA recommendations were included in the UDS with four action points linked to these.

#### Engagement with Māori

the single HIA recommendation, with a linked action point was included in the UDS. None of the Top Twenty Priority Actions deals explicitly with Māori development/concerns.

Other impacts of the HIA on local government policy were also described by key informants and workshop attendees. A key informant described that the HIA catalysed partnerships that contributed to amendments made to the Natural Resources Regional Plan (NRRP) [30].

Some policies were described to have had a direct and attributable link to the HIA (such as the proposal of a Christchurch City Health Development Plan, 2008) [31]. Other publications such as the Health Promotion and Sustainability Through Environmental Design Guide [32] and the CCC Community Charter [33] describe the UDS HIA as one of several contributing factors in their development. HIA as a tool has also been incorporated into the CDHB's 'Healthy Eating Active Living Action Plan' [34] as a result of the UDS HIA.

Several key informants described the HIA and its process as leading to a deeper consideration of health and inequalities in City Council policies. They described the HIA as giving permission for social and health principles to be included to a greater extent in CCC strategies and programmes.

### HIA impacts on cross-sectoral relationships (HIA Objective III)

*"We realised there was another whole sector that could swing in support of the UDS... to have health people there mucking in behind in support of densification and consolidated urban design option was really fantastic." *Senior Policy Maker, Environment Canterbury

Impact evaluation workshop participants and key informants described ongoing frequent contact between the key organisations which had been facilitated by relationships developed during the HIA. Local government and health board key informants described the HIA as facilitating different sectors in learning a new vocabulary and language. For example, where local government have talked of 'social development' and 'equity', health people would use the terms 'health outcomes' and 'health inequalities'.

Key informants described relationships built during the HIA as contributing to the launching of the electronic network, the South Island Public Health Analysis Information Base. This provides an interactive bulletin board, archive, and e-discussion for public health and local government throughout the South Island, with administration resting with Environment Canterbury and CDHB. Analysis of use of the SIPHAN information base on 8 April, 2008 showed there were 71 registered users and 152 posts, viewed a total of 1793 times.

Key informants in both local government and CDHB described the creation of a new public health physician post within local government as an impact attributable to the HIA. The HIA was considered to have given local government a new perspective on the skills and knowledge a public health person could provide as well as a clear track record that illustrated the benefits of input from a public health trained person. Serendipitous factors at play in particular included the support of a local government manager with core training in health as well as experience in management in the health sector and strong interest expressed in filling this role by the HIA project manager.

### HIA Impacts on engagement with Māori (HIA Objective IV)

*"It was not so much the event but the process of the HIA which has cemented relationships" *Māori consultation stream leader, CDHB

Ngāi Tahu had been invited to be part of the UDS Forum but their contribution and role prior to the process of the HIA was limited. The HIA Māori working group was led by a Māori public health physician who used the opportunity of the HIA to build relationships between the CDHB and Māori in Canterbury through meetings, hui and particularly through facilitating the visit of a Māori urban design specialist. Several key informants described the HIA's strong focus on Māori and felt this facilitated the subsequent re-engagement of Māori in the UDS Forum and final policy.

This engagement was illustrated with the participation of Ngāi Tahu in the Urban Forum, subsequent to the HIA, as well as the endorsement of the UDS by Mark Solomon, Kaiwhakahaere of Te Runanga o Ngāi Tahu (CEO of the Governing body of the Ngāi Tahu tribe) [27].

### HIA impacts on capacity and knowledge of HIA regionally and nationally (HIA Objective V)

"This has been the most ambitious HIA done in New Zealand to date – and what's more, the ambitious size and scope of it were matched by its delivery."

HIA consultant, Canterbury

Dissemination of the HIA results and process followed resulted in publications and presentations with national and international recognition of the HIA. Many of the key informants felt that this HIA had given credibility to the approach of HIA among local government policy makers both regionally and nationally. Key informants described this HIA as high profile and successful.

Key informants as well as workshop participants described what they saw as the success factors coming out of this HIA. The factors most frequently identified were strong leadership of the project and the creation of a public health physician position in the CCC and CDHB, jointly funded by both organisations. Several key informants reported that this nurtured many of the impacts of the HIA. This is the first joint appointment by a health board and local authority in New Zealand, and was described as a good example of the goodwill and effective relationships between health and local government developed through the HIA.

A third success factor described was strong cross-sectoral relationships. The entire HIA used collaborative process between CDHB, CCC and Environment Canterbury. Working and steering groups included members of different organisations and successful cross-sectoral relationships such as the CCC guide for Health Promotion and Sustainability [32] jointly launched by the Chief Executive Officer of the CDHB and the Mayor of Christchurch.

A fourth success factor described was the thorough documentation and dissemination of the HIA – which included writing up of the HIA in peer-reviewed journals [22,23] as well as the formal process evaluation [25]. The HIA report was described as being appropriately written and phrased to reflect organisational language and concerns without compromising the content and quality of recommendations. It was presented as a keynote presentation [35] in a conference in Melbourne and is used as a case study in HIA training in New Zealand [36] and Australia [37]. A key informant based in Australia described this HIA as an important milestone in the acceptance and credibility of HIA in Australasia.

*It 'feels' like we're bringing health into our planning more consistently and organically – especially at the CCC office level – but there's lots more to do at an executive and political level." *Programme Manager – CCC

Key informants described a change in ways of working attributable to the HIA. In the CDHB, HIAs are described as now being formally part of core business for the Division of Community and Public Health within CDHB. This HIA was felt to have catalysed a new focus on sustainability issues and for the CDHB Risk Management Committee to work on higher level policies. The HIA was considered to have led to further HIAs including the Central Plains Water Scheme (CPWS) HIA [38] and the Christchurch Transport Interchange project with CCC in 2008 [39].

Within the CCC, the HIA was described as a tool that has synergised well with the Local Government Act 2002 which provides the general framework and powers under which New Zealand's 85 democratically elected and accountable local authorities operate [40]. The Local Government Act recognises that local authorities are able to provide community governance at the local level and make a significant contribution to social, economic, environmental and cultural well-being. The CCC publication of the UDS HIA summary was used at the two-week technical Inquiry by Design workshops in August 2006 which used a principle-led approach to develop the settlement pattern of Greater Christchurch, considering social, cultural, environmental, growth and land use elements (p33) [27].

Many of those involved in policy making described increased understanding of health impacts. Examples of this include the use of the Healthy Housing Index on Council housing stock [41], in the Inquiry by Design workshops (August 2006), HIA training received by six CCC staff and work by the large cross-sectoral group of 'Healthy Christchurch' on a City Health Plan [31].

### Aspired impacts that the HIA did not realise

Several key informants and workshop participants described a strategic plan like the UDS as limited by regulatory mechanisms within New Zealand's Resource Management Act 1991 [42]. This limits the extent HIA can impact health determinants through limiting spheres of influence of local government policy, such as the UDS.

Universally, workshop attendees felt that greater dedicated human and financial resourcing for the HIA could have allowed the identification and appraisal of a broader range of determinants of health. For example, the 'waste' health determinant stream was withdrawn due to resource constraints. This would have provided a more comprehensive assessment of potential positive and negative, intended and unintended health impacts.

Several key informants felt that HIA as a practice has achieved minimal integration into the larger CDHB's core business. This theme recurred within the CCC, where HIA was considered to be useful and part of practice in some areas yet unheard of in others. Concern around lack of diffusion of HIA within organisations is repeated at the national level where disappointment was expressed that HIA has been used minimally by central government in New Zealand.

One key informant described inequalities as inadequately explored by the HIA process, with examples such as the differential equity impacts of urban densification on private outdoor space, and increased storm water run-off possibly leading to damp housing. A concern expressed at the workshop was that using a participatory HIA approach can increase the time required for policy formation, limiting potential responsiveness and agility of policy development processes.

### Future directions

A final section of the HIA impact evaluation workshop focussed on what should be done differently by partner organisations to achieve significant changes in health determinants in the future. Ideas arising from the workshop included:

• Requiring public organisations, such as local government and DHBs, to engage with strategic partner organisations before writing Long Term Council Community Plans (LTCCP) and District Annual Plans so that policy impacts on health determinants are considered routinely. This would embed HIA in these processes at an early stage, which it is not required for the end product;

• Leaders of key partner organisations invite other leaders to share in formation of strategic vision and planning. This could expand to make collaborative work requisite, such as a percentage of each employees time is allocated to work in another organisation to achieve mutual shared objectives, secondments, and the development of common public information portals e.g. linked Intranet for all public agencies.

## Discussion

The paper sets out the process and impact evaluations of the UDS HIA providing a valuable addition to the sparse area of HIA research. Evaluation indicates the HIA was carried out with an effective process, requiring moderate resources to achieve significant direct and indirect impacts. These impacts are related to: policy change and organisational culture change among organisations involved in the HIA; improved cross-sectoral relationships; and increasing recognition of the HIA tool more widely in Canterbury and New Zealand. There are a number of important issues arising from these evaluations including the cost-effectiveness of HIAs, the role of HIAs in cross-sectoral work, enabling factors to increase uptake of HIA recommendations by decision makers, possible factors limiting further HIA impacts as well as methodological issues with these evaluations.

### Cost of the HIA

Resource use is rarely described, but has been summarised in three papers [43-45]. Other HIA estimates of project time are 684 project hours [46] and 2784 project hours (assuming eight hours per day) [47] Relative to the other HIAs that have reported on time investment, the project hours of the UDS HIA are modest.

Costs for HIAs are calculated in diverse ways and these are infrequently described, however attempts to summarise costing have been performed [43-45,48]. The London Health Observatory provides an HIA cost calculator tool at the following URL . This would be one method to standardise cost calculation and allow legitimate comparison between HIAs. The method used for cost calculation in the UDS HIA was very similar to that used in this online calculator. A summary of costs of HIAs in Europe in the last decade ranged from $US 5720 to $US180325 depending on the level and type of HIA but excluding desktop HIAs [44]. Other HIA costing estimates in the UK vary from $US20000 [49] to $US157700 [43]. As details are not provided on how these costs were calculated, or the dimensions of the HIA, comparison is difficult.

Details of cost are included in this paper to provide transparent information about the resources investment and to assist future cost utility analyses of completed HIAs.

The sums in Table 3 suggest that the Christchurch HIA cost of $US42000 was moderate. It was performed with minimal direct funding (resources mostly 'in-kind'). The York study [45] sought to perform a cost – benefit analysis of HIAs. They were limited by the non-comparability of different HIAs (ranging from desk-top exercises to comprehensive primary data gathering), the difficulty in attributing impacts and outcomes specifically to HIAs and multiple factors extending the time and resources required for assessments. As others have suggested [43] HIAs are ill suited to formal cost-benefit analysis as many of the benefits of an HIA are indirect and in forms that cannot be easily measured. Summarising resource use described in the above papers, the costs of an HIA are linked primarily to the following factors: the dimensions of the HIA (rapid or comprehensive); the size of the issue being evaluated e.g. national versus local policy; the use of external consultants; and the extent of community consultation.

### Cross-sectoral collaboration

The importance of cross-sectoral collaboration described in key informant interviews and by workshop attendees is one of the key impacts of this HIA. It has facilitated further shared work, a joint position funded by health and local government and multiple examples of collaborative work. Cross-sectoral collaboration is a key indirect impact identified in other HIA papers [50-52]. However, despite such strong support, none of the three recommendations around intersectoral working groups in the HIA were included in the final UDS policy. Work for the first review of the UDS starts in 2009 and will look at general assumptions, institutional arrangements (including governance), and alignment with other agencies such as CDHB. This presents opportunities to review HIA recommendations.

Possible reasons for non-inclusion of the cross-sectoral recommendations are: the time and energy required for such groups to be representative and function well; feared loss of control by local government; regulatory restriction on cross-sectoral work (for example the RMA only permits public health authorities to submit on local authority regional policy via the Environment Court); and individual organisations' reporting constraints and tight timelines for projects meaning low priority for cross-sectoral work. These omissions in the UDS could lead to negative health impacts over time.

In New Zealand legislation (as elsewhere) there is a lack of clarity about which agencies have overall responsibility for determinants of health as they are intrinsically cross-sectoral in nature. Yet individual legislation such as the Land Transport Act 2003, the Building Act 2004 and the Local Government Act 2002 do include public health objectives [53]. These provide incentives to some sectors to consider health impacts and determinants [54]. The new Public Health Bill also seems to provide increased opportunity for closer coordination between local government and public health authorities and could provide an institutional platform for HIA [53].

The potential for HIA as a bridge to increase cross-sectoral work has been increasingly recognised internationally and by the key players in this HIA. An analysis of a container port development that used a cross-sectoral approach [55] found benefits of: opening up contacts and working relationships within each organisation; shared expertise; opportunities to address a wider range of health impacts; pooled resources; and increased credibility with decision makers. Others have also described the importance of these indirect impacts of HIA [50,56] and particularly the links between research and decision making where the HIA process impacts on decision makers to increase community perspectives and health determinants, leading to pro-health policy.

### Enabling factors for inclusion of HIA recommendations into the UDS

Over two thirds (24 of 33) of the HIA recommendations were included in the final UDS policy. This HIA included all the enablers related to decision makers and policy process described by Davenport et al in their review of enablers in 88 HIAs [51]. These include: involvement of decision makers in conduct and planning of the HIA (they were present on both HIA steering and working groups); clear organisational commitment to HIA (dedicated HIA staff time and resources); the subject of the HIA not being controversial; tailored presentation of report and recommendations to reflect organisational concerns; and provision of realistic recommendations, some of which concur with other political drivers at the time (for example, concerns of Peak Oil and rising petrol prices in 2005 linked well to HIA Transport stream recommendations around Active Transport). The success factors of the Christchurch HIA identified during the impact evaluation workshop were leadership, dedicated resources, cross-sectoral relationships and thorough, tailored documentation and recommendations.

### HIA recommendations not included in the UDS

The absence of a health determinants focus in the Twenty Priority Actions of the UDS suggests that monitoring health determinants and outcomes is not considered a key responsibility of local authorities. Some local government planners argue health indicators are a sub-set of social indicators, yet explicit inclusion of health indicators could increase focus on health outcomes.

HIA has been described as particularly effective at increasing understanding of health determinants in local government settings in Australia [57] and New Zealand [58]. The effectiveness of the HIA to increase consideration of health determinants generally among urban planners has been described by many others [51,55,57,59]. In New Zealand the value of HIA in local government has been supported by statutory obligations of the Local Government Act 2002 which requires promotion of social, cultural, economic and environmental well-being.

This paper finds that the UDS HIA was an important tool which has supported changes in planning and policy among partner organisations, but also describes that impacts have been less than hoped for. Lack of diffusion of HIA within and across larger organisations was reported by CCC and CDHB, yet other informants suggest significant gains were achieved with this single HIA and subsequent HIAs in the region promise to build on this base. Others have described HIA uptake in "silos" in local government but suggest that effective HIAs lead to wider dissemination within organisations [57].

The concern expressed at the impact evaluation workshop that HIA can reduce responsiveness of policy making and increase time-frames for policy development has been described elsewhere [50,52,57]. Yet if HIAs are initiated and completed with appropriate time-frames in the policy or project development cycle, this may not be a problem. The UDS HIA was performed and reported in a timely fashion that did not slow policy making and its process in fact orchestrated consultation and generated data through both the Māori and social connectedness working groupss that were useful for the UDS policymakers.

Working party members described that while the HIA itself had strong leadership (facilitated by a strong steering committee with representation from local government and health), leaders of the HIA's partner organisations also needed conviction and enthusiasm for the HIA to achieve greater adoption. A review of ways that HIA can be made most useful to local government in New South Wales (NSW) echoes this. The author suggests selecting the best HIA steering committee and overcoming 'silos' in councils as two factors for HIA success in local government [57]. This review supports the importance of HIA as an advocacy tool to change priorities to support health and improved relationships between health and local government.

### Limitations of this evaluation

This evaluation used a multi-method qualitative methodology to gather information on the impacts of the HIA. The depth and breadth of the analysis was potentially limited by the unavailability of three identified key informants for interview and competing work priorities limiting participation at workshops. The evaluator was an employee of CDHB at the time of the process evaluation, which may have influenced the candour of participants and therefore influenced the analysis.

## Conclusion

The HIA of the Draft Greater Christchurch UDS was broadly successful and effective with significant direct and indirect impacts. It strengthened cross-sectoral partnerships which have led onto further initiatives, including a new position created for a Public Health Physician in the Christchurch City Council, and work commenced by CDHB and CCC on a City Health Plan and subsequent HIAs.

This HIA has contributed to a more prominent role for health on the local government agenda and improved knowledge of a social determinants model of health by non-health professionals and the public. Significantly, the majority of the HIA recommendations have been adopted by the policy body – the Greater Christchurch Urban Development Forum. The HIA process has also contributed to policy implementation such as amendments to the NRRP [30]. There has been improved engagement and relationship with Māori, illustrated through improved consultation mechanisms between health, local government and Māori.

Permeation of a health determinants approach (as provided by the HIA tool) into the tissue of the larger UDS partner organisations is yet to happen. Currently 'health' organisations continue to be dominated by health services provision with little focus on changing health determinants. Ongoing and even greater commitment to changing health determinants by health and local government is essential to achieve long-term health outcomes.

The inclusion of the health determinants defined in this HIA into the long-term Greater Christchurch UDS increases opportunity for a healthy urban environment, and therefore healthy citizens of the Greater Christchurch area. This paper adds to the limited literature that evaluates HIAs. It evaluates the UDS HIA as a generally highly successful process, with significant impacts that achieved its purposes. This in turn can increase the confidence of policy makers and funders in the value HIA as a tool that can lead to improved health for the community.

## Abbreviations

CCC: Christchurch City Council; CDHB: Canterbury District Health Board; HIA: Health Impact Assessment; UDS: Urban Development Strategy.

## Competing interests

The authors declare that they have no competing interests.

## Authors' contributions

KM responsible for methods, primary research and first draft of paper and revision of subsequent drafts. BHR supported with literature review, drafting of the paper and revision of subsequent drafts.

## Pre-publication history

The pre-publication history for this paper can be accessed here:


